# Pembrolizumab‐Induced Chronic Rhinosinusitis in a Patient With Non‐Small Cell Lung Cancer

**DOI:** 10.1002/rcr2.70362

**Published:** 2025-10-05

**Authors:** Yuji Yamasaki, Hiroshi Ohnishi, Masahiro Komori, Junya Mizuta, Mitsuko Iguchi, Akihito Yokoyama

**Affiliations:** ^1^ Department of Respiratory Medicine and Allergology Kochi Medical School, Kochi University Nankoku Japan; ^2^ Department of Integrative Respiratory Medicine Ehime University Graduate School of Medicine Toon Japan; ^3^ Department of Otolaryngology, Head and Neck Surgery Kochi Medical School, Kochi University Nankoku Japan; ^4^ Department of Diagnostic Pathology, Kochi Medical School Kochi University Nankoku Japan

**Keywords:** chronic rhinosinusitis, immune checkpoint inhibitor, immune‐related adverse event, non‐small cell lung cancer, pembrolizumab

## Abstract

A 63‐year‐old man was administered pembrolizumab for stage IV squamous cell lung cancer. After 54 cycles of pembrolizumab, the patient maintained complete remission of lung cancer but developed nasal obstruction, olfactory disturbance, conjunctivitis, stomatitis, glossitis, pharyngitis, and weight loss. Laboratory data showed increased levels of neutrophils, eosinophils, C‐reactive protein, squamous cell carcinoma antigen, and cytokeratin 19 fragments. Sinus computed tomography revealed obstruction of the maxillary, ethmoid, and sphenoidal sinuses. Histological examination of nasal septal tissue revealed chronic rhinosinusitis with infiltration primarily of eosinophils, neutrophils, CD3^+^CD8^+^ T lymphocytes, and CD20^+^ B lymphocytes, along with destruction of nasal epithelium and glands. The patient was diagnosed with immune‐related chronic rhinosinusitis and treated with oral prednisolone after discontinuation of pembrolizumab. Herein, we report a rare case of pembrolizumab‐induced chronic rhinosinusitis without nasal polyps that was successfully treated with oral corticosteroid therapy.

## Graphical abstract

We report a rare case of immune‐related chronic rhinosinusitis that developed in a patient with stage IV squamous cell lung cancer who achieved complete remission after 54 cycles of pembrolizumab. Histological analysis revealed predominant CD8^+^ T lymphocyte infiltration with epithelial destruction in nasal tissue. Follow‐up sinus CT performed 3 months after corticosteroid treatment showed marked improvement in sinus obstruction.

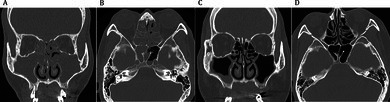

## 1 Introduction

Immune checkpoint inhibitors (ICIs), including the anti‐programmed cell death protein 1 (PD‐1) inhibitor pembrolizumab, enhance antitumor activity by blocking the PD‐1/programmed cell death ligand 1 (PD‐L1) pathway between T lymphocytes and tumour cells. However, activation of CD8^+^ T lymphocytes can lead to immune‐related adverse events (irAEs) in various organs. Chronic rhinosinusitis (CRS) was recently reported as a rare irAE [1, 2, 3].

## 2 Case Report

A 63‐year‐old man was treated with four cycles of first‐line combination chemotherapy with carboplatin, nab‐paclitaxel, and pembrolizumab, followed by maintenance ICI therapy with pembrolizumab 200 mg every 3 weeks for stage IV squamous cell carcinoma (SCC) of the left upper lung lobe. After 54 cycles of pembrolizumab, the patient remained in complete remission of lung cancer but presented with nasal obstruction, olfactory disturbance, conjunctivitis, stomatitis, glossitis, pharyngitis, and weight loss. No dysphagia or xerostomia was observed after the initiation of pembrolizumab therapy. He had a smoking history of 30 pack‐years, did not consume alcohol, and had no history of bronchial asthma or rhinosinusitis.

**FIGURE 1:**
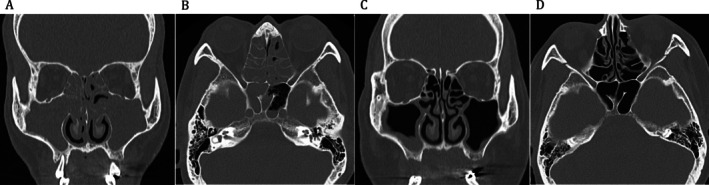
Coronal (A) and axial (B) views of computed tomography (CT) images of the sinuses at initial presentation show obstruction of the maxillary, ethmoidal, and sphenoidal sinuses. Coronal (C) and axial (D) views of CT images of the maxillary and ethmoidal sinuses 3 months after prednisolone treatment show improvement of chronic rhinosinusitis.

**FIGURE 2:**
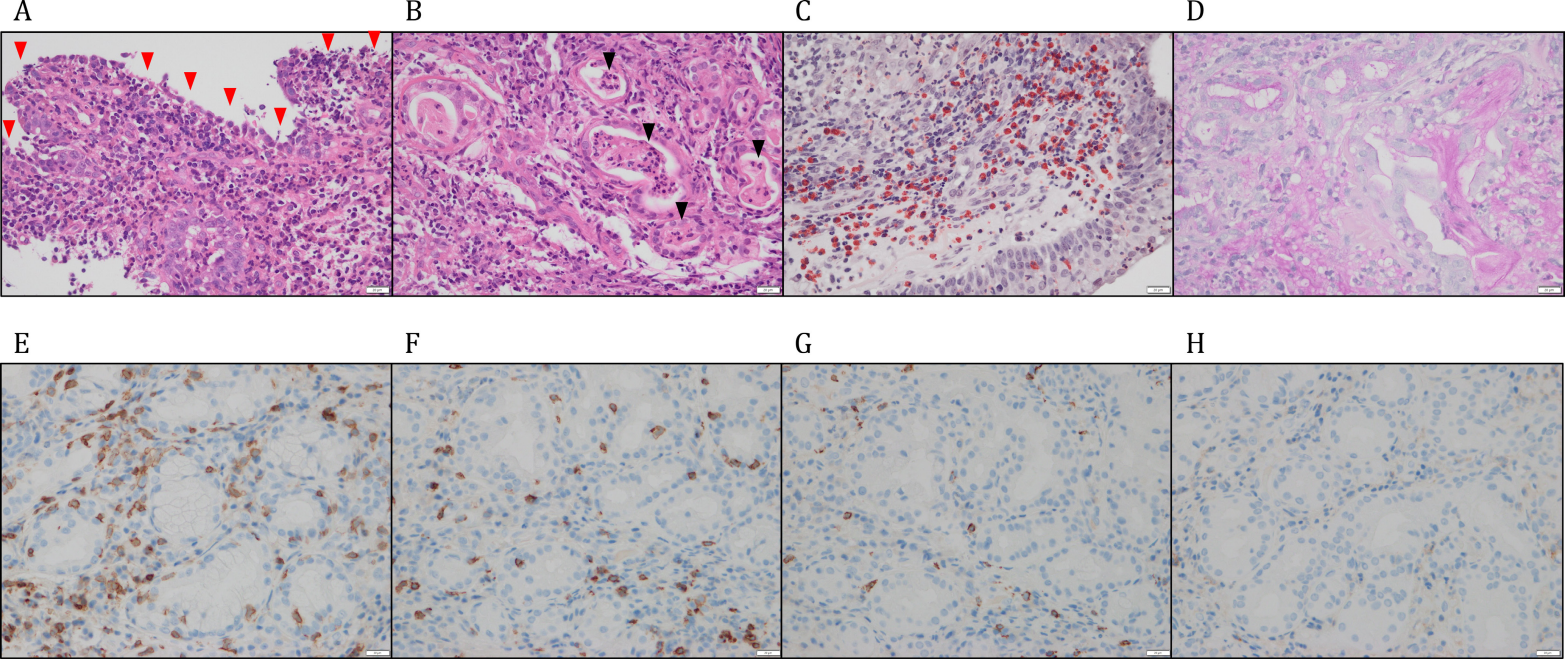
Histological examination of a left nasal septum biopsy specimen shows erosion and ulceration of the nasal epithelium (A, red arrowheads); accumulation of neutrophils in nasal gland lumina forming microabscesses (B, arrowheads); infiltration of many eosinophils into the nasal submucosa (C, orange‐stained cells); and leakage of mucus into the extraglandular interstitium (D, purple‐stained substances), suggesting destruction of mucosal and glandular structures of the nasal septum. Immunohistochemistry shows infiltration mainly of CD3^+^CD8^+^ T lymphocytes (E, F) and CD20^+^ B lymphocytes (G), with fewer CD4^+^ T lymphocytes (H), confirming chronic rhinosinusitis due to immune‐related mechanisms. (A, B) Haematoxylin–eosin; (C) direct fast scarlet 4BS; (D) periodic acid–Schiff; (E) anti‐CD3 mAb; (F) anti‐CD8 mAb; (G) anti‐CD20 mAb; and (H) anti‐CD4 mAb staining (original magnification ×200; bars indicate 20 μm).

Laboratory examination showed neutrophilia (10,470/μL), eosinophilia (maximum 1270/μL, 12.7%), and increased levels of C‐reactive protein (8.09 mg/dL), SCC antigen (7.4 ng/mL, normal < 1.5 ng/mL), and cytokeratin 19 fragment (CYFRA; 3.8 ng/mL, normal < 3.5 ng/mL). Morning serum cortisol (19.2 μg/dL), immunoglobulin E (90.8 IU/mL), and IgG4 (27.7 mg/dL) levels were within normal ranges. Cytoplasmic and perinuclear antineutrophil cytoplasmic antibodies were negative. ^18^F‐fluorodeoxyglucose positron emission tomography‐computed tomography (CT) showed increased ^18^F‐fluorodeoxyglucose accumulation in the oral cavity, pharynx, sinuses, salivary glands, and the entire oesophagus. Sinus CT revealed obstruction of the maxillary, ethmoidal, and sphenoidal sinuses (Figure 1A,B). The JESREC score [4] was 13 points (three points for bilateral disease, two points for ethmoid sinus disease, and eight points for blood eosinophilia), suggesting the possibility of eosinophilic CRS (ECRS). Fiberscopic examination of the nasal cavity revealed crusting but no nasal polyps (data not shown). Histological examination of the left nasal septal tissue revealed CRS with infiltration primarily of CD3^+^CD8^+^ T lymphocytes, CD20^+^ B lymphocytes, neutrophils, and eosinophils, along with destruction of the nasal glands (Figure 2A–H). Upper gastrointestinal endoscopy showed no evidence of oesophageal cancer; however, characteristic rings and longitudinal grooves were suggestive of eosinophilic esophagitis (data not shown).

The patient was diagnosed with immune‐related CRS and treated with oral prednisolone 45 mg/d (1 mg/kg body weight) after discontinuation of pembrolizumab. The patient's nasal symptoms and taste disorder gradually resolved; body weight increased; serum levels of C‐reactive protein, SCC, and CYFRA normalised; and corticosteroids were tapered off. Sinus CT performed 3 months after corticosteroid treatment showed marked improvement in sinus obstruction (Figure 1C,D). One year and 10 months after pembrolizumab discontinuation and 11 months after tapering off corticosteroids, the patient remained in complete remission of lung cancer and showed no recurrence of CRS or eosinophilic esophagitis.

## 3 Discussion

This case demonstrates that CRS should be considered even in rare manifestations of irAEs. In the US Food and Drug Administration Adverse Event Reporting System database, nasal and sinus irAEs accounted for 0.62% (826 cases) of 133,118 irAE cases [2]. The reported cases of CRS due to irAEs with PD‐1 inhibitors included one case of ECRS, seven cases of CRS with nasal polyps, and 13 cases of CRS without nasal polyps. Among the cytotoxic T‐lymphocyte‐associated protein 4 inhibitors, one case of ECRS and one case of CRS were reported. To date, no cases associated with PD‐L1 inhibitors have been documented. In this case, sinus CT showed features consistent with CRS, and blood eosinophilia was present, making the differential diagnosis of CRS difficult without a nasal biopsy. Immunohistochemistry with anti‐CD4 and anti‐CD8 monoclonal antibodies is useful for diagnosing irAE‐associated CRS. The presence of blood eosinophilia, CRS with infiltration of eosinophils and CD8^+^ T lymphocytes, and eosinophilic esophagitis suggested that the mechanism in this patient may have been driven by CD8^+^ T lymphocytes and type 2 inflammation.

The management of irAEs typically includes temporary discontinuation of ICIs and/or systemic corticosteroids in cases of severe inflammation. Because the patient presented with relatively severe irAEs, including weight loss and impaired quality of life, pembrolizumab was discontinued, and corticosteroids were administered. The patient remained in complete remission for 1 year and 8 months after pembrolizumab discontinuation. Permanent cessation of ICIs may be necessary in severe cases; however, this decision must be carefully balanced against the need for ongoing cancer treatment. If lung cancer recurs, an anti‐PD‐L1 inhibitor may be considered as an alternative, as no CRS cases have been reported in association with PD‐L1 inhibitors [2].

The KEYNOTE‐024 trial protocol for PD‐L1‐positive non‐small‐cell lung cancer specified pembrolizumab administration for up to 35 cycles [5]. We initially proposed discontinuing pembrolizumab after completing 35 cycles. However, because the patient had experienced rapid progression of lung cancer before treatment and was concerned about recurrence after discontinuation of pembrolizumab, the patient chose to continue therapy. Treatment was therefore continued, and no irAEs were observed through 53 cycles. Evidence regarding the efficacy and/or safety of pembrolizumab maintenance therapy beyond 35 cycles is lacking; therefore, caution is warranted for long‐term administration.

Because of the elevated serum levels of SCC antigen and CYFRA in this patient, we initially considered recurrence of SCC of the lung or *de novo* development of other SCCs, such as oesophageal or head and neck cancers. However, no recurrence of SCC was found, and instead, CRS was observed. Corticosteroid therapy improved CRS and normalised serum levels of SCC and CYFRA, indicating that ICI‐induced CRS, accompanied by eosinophilic esophagitis, may elevate these markers, possibly due to squamous epithelial damage. SCC has been reported to increase in benign dermatologic diseases, such as atopic dermatitis, and type 2 cytokines, such as interleukin‐4 and ‐13, can induce SCC antigen expression in cultured keratinocytes, thereby increasing serum SCC levels [6].

In conclusion, CRS caused by ICIs is a rare but potentially significant contributor to diminished quality of life and should be considered in the differential diagnosis of irAEs.

## Author Contributions

Y.Y., H.O., M.K., J.M., and A.Y. cared for the patient. M.I. performed the pathological diagnosis. All authors contributed to writing and approved the final version of the manuscript. These images have not been published previously.

## Consent

The authors declare that appropriate written informed consent was obtained for the publication of this manuscript and the accompanying images using the Journal form.

## Conflicts of Interest

H.O. receives lecture fees from MSD K.K.

## Data Availability

Data sharing not applicable to this article as no datasets were generated or analysed during the current study.
